# Current status of molecular-targeted drugs for endometrial cancer (Review)

**DOI:** 10.3892/mco.2013.140

**Published:** 2013-06-26

**Authors:** YUYA NOGAMI, KOUJI BANNO, IORI KISU, MEGUMI YANOKURA, KIYOKO UMENE, KENTA MASUDA, YUSUKE KOBAYASHI, WATARU YAMAGAMI, HIROYUKI NOMURA, EIICHIRO TOMINAGA, NOBUYUKI SUSUMU, DAISUKE AOKI

**Affiliations:** Department of Obstetrics and Gynecology, School of Medicine, Keio University, Shinjuku-ku, Tokyo 1608582, Japan

**Keywords:** endometrial cancer, molecular-targeted drugs, hMLH1, bevacizumab, gefitinib, trastuzumab, mammalian target of rapamycin inhibitors

## Abstract

Endometrial cancer is a common gynecological malignant tumor in Western countries and its incidence has also been on the increase in Asia. Genetic abnormalities related to onset and progression of malignancy in the endometrial membrane and signaling system have been identified and the developmental mechanism of endometrial cancer is becoming elucidated. The identification of the molecules related to these abnormalities has led to new potential treatment regimens for endometrial cancer, using molecular-targeted drugs. The current chemotherapy for endometrial cancer often causes systemic side effects that require discontinuation of the treatment. Furthermore, a treatment regimen for cancers of rare histological types has not been established. Recent studies on endometrial cancer revealed patterns of genetic disorders that differ among the histological types. Genetic and molecular information that underlie pathological changes and is associated with DNA mismatch repair genes and epigenetic regulation was also identified. Targeting of these mechanisms with molecular-targeted drugs has been performed with the aim of linking treatment to the carcinogenic mechanism at the molecular and genetic levels. However, the response rates with single-agent therapy are generally low and several problems remain unresolved. Trials of combinations of molecular-targeted drugs with currently available treatments and identification of factors determining sensitivity are required to overcome these difficulties.

## 1. Introduction

Endometrial cancer is a common gynecological malignancy in Western countries and is fourth in incidence after breast, lung and colon cancer. The incidence of endometrial cancer has increased by 21% since 2008 and the mortality rate per 100,000 cases has increased by ≥100% over the last two decades and by 8% since 2008 (1). The incidence of endometrial cancer in the Japanese population has also increased, with cases of endometrial cancer accounting for ~10% of all uterine cancers in the 1970s, 42% in 1998, 47% in 2002 and 51% in 2008 (2).

This background has led to studies on gene abnormalities related to the onset and progression of endometrial malignancy and the results of these studies have gradually revealed the developmental mechanism of endometrial cancer. The development of a malignant tumor generally requires an accumulation of genetic mutations through a process referred to as ‘multistep carcinogenesis’. In the endometrium, genetic and epigenetic abnormalities have been described at each step leading from a precursor lesion to invasive cancer. Several molecules associated with these abnormalities have been identified and novel treatment regimens for endometrial cancer are being developed based on drugs targeting these molecules, i.e., molecular-targeted drugs.

The efficacy of conventional anticancer drugs depends on their toxic effects on cancer cells and the mechanism of action is often determined after their introduction to clinical use. The cytotoxicity of current anticancer drugs also applies to normal cells and the achievement of therapeutic effects may be associated with severe adverse events. Some of these side effects, such as bone-marrow suppression, mucosal damage in the digestive tract and hair loss, may be independent of the drug class, whereas others are specific to particular anticancer drugs. Thus, chemotherapy is performed as a balance of therapeutic and adverse effects. By contrast, molecular-targeted drugs act by specifically targeting the mechanisms of cancer cell proliferation and metastasis at the molecular level. The targeting of specific molecules in cancer cells may achieve therapeutic effects with fewer side effects. Currently, several drugs are being developed against various molecules that were identified to play key roles in the molecular mechanisms related to carcinogenesis.

## 2. Current status of endometrial cancer treatment

Endometrial cancer is predominantly diagnosed pathologically and the prediction of prognosis and treatment planning are performed based on pathological findings and evidence from clinical trials. Preoperative diagnosis is based on histological examination by endometrial biopsy and imaging using magnetic resonance imaging (MRI) and computed tomography. The level of serum CA-125, a tumor marker, is also measured. However, serum CA-125 is only elevated in ≤60% of endometrial cancer patients and its clinical utility is limited (3). Evaluation of gross myometrial invasion is important, since it is associated with the risk of lymph node metastasis and mortality in endometrial cancer (4). However, in 116 patients with histologically diagnosed endometrial cancer, Nakao *et al*(5) demonstrated that preoperative MRI had an accuracy of only 62.1% for the evaluation of myometrial invasion. Histological diagnosis is also of limited value for the detection of microinvasive cancer and is less objective and variable among facilities and countries. Thus, accurate preoperative diagnosis is challenging in endometrial cancer and the stage is often determined postsurgically, based on the International Federation of Gynecology and Obstetrics staging system.

Treatment of endometrial cancer is planned based on age, presence of complications, extent of the lesion and the characteristics of the cancer. Surgery is the first-line treatment, with the objectives of complete elimination or size reduction of the tumor for improving prognosis, determination of stage and facilitation of additional treatment options. Surgical treatment and postoperative adjuvant therapy were determined based on evidence from clinical trials, although standard protocols have not been established.

The standard chemotherapy for endometrial cancer currently used worldwide is a combination of doxorubicin and cisplatin (AP therapy). The Gynecologic Oncology Group (GOG) 122 trial in advanced (stage III or IV) endometrial cancer demonstrated an improved prognosis with AP therapy and the value of adjuvant therapy in endometrial cancer. However, 17% of patients had to discontinue the chemotherapy due to side effects (6). The side effects of AP therapy may be hematological, including grade 3/4 white blood cell toxicity (55%) or non-hematological, including grade 3/4 alopecia (72%) and nausea/vomiting (36%) (7). A combination of taxanes and platinum-containing drugs (TC therapy) has also been used as adjuvant therapy and chemotherapy for advanced or recurrent endometrial cancer. For example, a trial of TC therapy with paclitaxel and carboplatin for advanced or recurrent endometrial cancer achieved overall response rates of 63 (8) and 87% (9), respectively. The side effects of TC therapy include grade 3/4 hematological toxicities, such as leukopenia (61.7%), anemia (21.7%) and thrombocytopenia (5.0%) and grade 3 non-hematological toxicities, including neuropathy (5.0%), nausea (3.3%) and myalgia (6.7%) (10). Thus, the side effects of chemotherapy are frequent and continuation of treatment may be challenging for patients in poor systemic condition. In addition, standard treatment protocols for several cancers of rare histological types have not been established, due to the time required for accumulation of evidence on efficacy.

The current problems in the treatment of endometrial cancer may be resolved by molecular-targeted drugs, by specifically targeting carcinogenic mechanisms established at the molecular and genetic levels. These drugs target cancer-specific molecules, which may reduce systemic side effects and potentiate the screening of targeting molecules and the development of drugs for cancers of rare histological types.

## 3. Genetic mutations associated with the development of endometrial cancer

Numerous genetic mutations have been linked to the development of endometrial cancer. Cancer development generally requires three steps: initiation of carcinogenesis (initiation); promotion as a single clone (promotion); and establishment of invasive capability (progression). In endometrial cancer, the mutation of genes associated with cancer initiation vary with clinical characteristics (type I or II), tissue differentiation and histological type. For example, endometrioid adenocarcinoma, a type I endometrial cancer, exhibits rates of *K-Ras* and p53 mutations of 26 and 17%, respectively, whereas in serous adenocarcinoma, a type II endometrial cancer, these rates are 2 and 93%, respectively (11). Similarly, in clear cell adenocarcinoma, a particular histological type of endometrial cancer, the levels of estrogen receptor (ER), progesterone receptor (PR) and Ki-67 are similar to those in serous adenocarcinoma while p53 is significantly lower, whereas ER and PR are significantly lower, Ki-67 is significantly higher and p53 tends to be higher compared to endometrioid adenocarcinoma (12). Thus, the patterns of genetic mutations and the developmental mechanisms may vary among histological types.

These genetic mutations do not occur randomly, but arise due to defects in the repair mechanisms. DNA typically has a mutation rate of 1/10^7^ replications, which is subsequently repaired. However, genetic mutations accumulate if DNA repair mechanisms are defective. The genes associated with DNA repair are referred to as DNA mismatch repair genes and include *human mutS homolog 2 (hMSH2)* and *human mutL homolog 1 (hMLH1)*. These genes are causative of the Lynch syndrome, which is associated with colon, endometrial, ovarian and gastric cancer on a familial basis. Microsatellite DNA repeats are repeating sequences of a few bases, such as CA and CAG, that are widespread in the human genome. Instability of genes resulting from failure to repair errors in these domains during DNA replication is referred to as microsatellite instability (MSI). MSI is a genetic disorder that is strongly associated with the generation of endometrial cancer and is particularly common in endometrioid adenocarcinoma (13).

## 4. Epigenetic abnormalities associated with the development of endometrial cancer

The DNA mismatch repair genes *hMLH1* and *hMSH2* are strongly associated with the development of type I endometrial cancer, although mutations of these genes occur at a low level (14). However, a reduced expression of the hMLH1 protein occurs due to hypermethylation of the *hMLH1* promoter (15), indicating that epigenetic mutations are important in endometrial carcinogenesis. Kanaya *et al*(16) demonstrated that >80% of the CpG sites were methylated in the *hMLH1* promoter in ~30% of cases of endometrioid adenocarcinoma, resulting in a reduced expression of the *hMLH1* protein. Since these changes were also detected at high rates (~40%) in the normal tissue surrounding the tumor, it was concluded that hypermethylation of the *hMLH1* promoter occurs at an early stage of endometrial cancer (16). This finding indicates that changes in genes and molecules may lead to morphological changes. For example, the initial hypermethylation of the *hMLH1* promoter may reduce the abillity to repair mismatches during DNA replication, which may then lead to mutations of *phosphatase and tensin homolog (PTEN)* and subsequent generation of endometrioid adenocarcinoma. Similar hypermethylation of the *hMLH1* promoter, MSI and *PTEN* mutations have not been detected in serous adenocarcinoma, a type II endometrial cancer. However, p53 mutations have been detected in ≥90% of cases of serous adenocarcinoma (17), indicating that this may be the disease-causative mutation.

Various mutations in pathways associated with generation of endometrial cancer have been described, including *K-Ras* in the Raf/MEK/ERK pathway (18). *K-Ras* mutations are detected in ~30% of endometrioid adenocarcinomas of all histological grades and in 15% of cases of endometrial hyperplasia and are likely to be associated with endometrial carcinogenesis. However, *K-Ras* mutations are detected in only 2% of serous adenocarcinomas (11). Other molecules associated with endometrial cancer include β-catenin, which is crucial in regulating Wnt signaling and cell adhesion (19) and adenomatous polyposis coli protein, the expression of which is reduced by promoter methylation in endometrioid adenocarcinoma (20).

## 5. Molecular-targeted drugs for endometrial cancer

Various pathways are involved in the development of endometrial cancer and several genes are associated with each pathway. Differences in genes and molecules associated with carcinogenesis determine cancer characteristics and considering these differences may be important for cancer treatment in the future.

Treatment targeting the general characteristics of cancer includes antiangiogenic agents, such as bevacizumab, aflibercept and thalidomide. Bevacizumab is a humanized monoclonal antibody against vascular endothelial growth factor (VEGF)-A. VEGF is a cytokine associated with the promotion of cell division and permeability of vascular endothelial cells. VEGF is essential for normal angiogenesis and exhibits enhanced expression in cancer cells. Under conditions of oxygen and nutrient excess, VEGF enhances angiogenesis and promotes the proliferation and metastasis of cancer cells. In endometrial cancer, the expression levels of VEGF have also been associated with prognosis (21). In a phase II trial of single-agent bevacizumab in recurrent endometrial cancer, 7 of 52 patients (13.5%) exhibited a response [complete response (CR), 1 and partial response (PR), 6] and 21 patients (40.4%) had a progression-free survival (PFS) of at least 6 months. The median PFS was 4.2 months and the overall survival was 10.5 months. The adverse reactions were the same as those with conventional bevacizumab therapy, without reported perforation of the digestive tract and fistula formation (22). The GOG 229-E phase II trial of bevacizumab in cases with distant metastasis of endometrial cancer is ongoing (23). Aflibercept (VEGF Trap-Eye) is a fusion protein that exhibits high-affinity binding to VEGF-A, VEGF-B and placental growth factor and thus has a unique mechanism of action as an inhibitor of angiogenesis. In a phase II trial of aflibercept in recurrent endometrial cancer, 3 of 44 patients (6.8%) exhibited a response (CR, 0 and PR, 3) and 18 (41.0%) had a PFS of 6 months; however, of these 18 patients, 8 had to discontinue aflibercept due to toxicity and initiate another therapy before 6 months had elapsed. The median PFS was 2.9 months and the overall survival was 14.6 months (24). Thalidomide has a plurality of antitumor properties, including an anti-angiogenetic action, although the precise mechanism has not been elucidated. In a phase II trial in persistent or recurrent endometrial cancer refractory to chemotherapy, 3 of 24 patients (12.5%) exhibited a response (CR, 0; PR, 3) and 2 (8.3%) had a PFS of ≥6 months. The median PFS was 1.7 months and the overall survival was 6.3 months (25).

Targeting of molecules in the carcinogenic pathways of endometrial cancer includes the use of human epidermal growth factor receptor (EGFR) inhibitors, such as gefitinib, erlotinib and cetuximab. EGFR is a transmembrane protein comprising an extracellular EGF-binding domain and an intracellular protein tyrosine kinase domain. EGFR is expressed in normal endometrial membrane and its overexpression is associated with the stage of endometrial cancer and a poor prognosis (23). Gefitinib and erlotinib are low-molecular-weight tyrosine kinase inhibitors. In a phase II trial of gefitinib for recurrent or persistent endometrial cancer, 1 patient had a CR and the response rate was 3.4% (26). In a similar phase II trial of erlotinib in 23 patients with recurrent endometrial cancer, 1 patient had a PR and the response rate was 4.3% (27). Cetuximab is a monoclonal antibody against EGFR. In a preclinical study, Takahashi *et al*(28) observed that cetuximab administered against human endometrial cancer transplanted in nude mice inhibited cancer cell proliferation, peritoneal metastasis and lymph node and lung metastasis *in vivo*, prolonging host survival. A phase II trial of cetuximab in patients with advanced or recurrent endometrial cancer is ongoing.

Trastuzumab and lapatinib are human EGFR type 2 (HER2)-related inhibitors that affect signal transduction. Trastuzumab is a monoclonal antibody against the extracellular domain of HER2 and is currently the standard treatment for HER2-positive breast cancer. A phase II trial of single-agent trastuzumab against advanced or recurrent endometrial cancer did not demonstrate activity against endometrial cancers with HER2 overexpression (29). However, this may be attributed to problems in the study design (30). A case report demonstrated that trastuzumab may be effective against endometrial cancer and this has led to a review of the activity of trastuzumab (31). Certain serous adenocarcinomas overexpressing HER2 and trastuzumab may be effective for this type of endometrial cancer (31). Lapatinib is an inhibitor targeting EGFR and HER2 that is currently being evaluated in the GOG 229-D phase II trial for recurrent endometrial cancer.

The effects of *PTEN* mutations in type I endometrial cancer may be reduced by mammalian target of rapamycin (mTOR) inhibitors, such as temsirolimus and ridaforolimus, by blocking the phosphoinositide 3-kinase/AKT/mTOR pathway. In a phase II trial of temsirolimus as first-line treatment for recurrent endometrial cancer previously untreated with chemotherapy, 5 of 19 patients (26%) had a PR and 12 (63%) had stable disease (SD) (32). In a phase II trial of temsirolimus as second-line treatment for recurrent endometrial cancer that had been previously treated with chemotherapy, the PR and SD rates were 7 and 44%, respectively (33). With ridaforolimus, 13 of 45 patients (28.9%) achieved a clinically beneficial response (CR, PR or SD) for ≥16 weeks (34). Based on these results, the National Cancer Institute of Canada and the Gynecologic Cancer Intergroup aim to perform a phase III trial of ridaforolimus.

The response rates of molecular-targeted drugs based on completed phase II trials are presented in Table I. These rates are relatively high for mTOR inhibitors, although they are generally lower compared to 43.3% (7) or 60.0% (35), previously reported for AP therapy and 63% (8) or 87% (9) previously reported for TC therapy for advanced or recurrent endometrial carcinoma, respectively, suggesting that there are several problems to be addressed regarding the use of molecular-targeted drugs. These include the multiplicity of carcinogenetic pathways and associated genes, rendering inhibition of a single molecule insufficient for anticancer activity. Thus, development of drugs with a plurality of targets, use of combinations of molecular-targeted drugs and use of additive and synergistic effects through combination with current anticancer agents or hormone drugs may be required.

Drugs with a plurality of targets include sunitinib, brivanib, sorafenib and imatinib. Sunitinib is a multi-kinase inhibitor that is currently under clinical trials to assess its effectiveness against recurrence or metastasis of endometrial cancer. Brivanib is a multi-targeted tyrosine kinase inhibitor that is under evaluation in the GOG 229-I phase II trial. Sorafenib is a tyrosine kinase inhibitor with an antiangiogenic action that was evaluated in a phase II trial group at the University of Chicago (36). Imatinib is a tyrosine kinase inhibitor targeting *Abl*, *c-kit* and platelet-derived growth factor receptor (PDGFR) that is currently in a phase II trial in combination with paclitaxel for serous adenocarcinoma with high expression of *Abl* and PDGFR. mTOR inhibitors are also under evaluation in several clinical trials in combination with other therapies. These include the GOG 229-G clinical trial of a combination of bevacizumab and temsirolimus and the GOG 248 randomized phase II trial of single-agent temsirolimus and a combination of hormone therapy and temsirolimus. The results of these trials are expected to be of particular interest, since single-agent therapy with mTOR inhibitors has achieved relatively high response rates.

Histone deacetylase inhibitors (HDACIs) are potential multi-target molecular-targeted drugs that may enhance the expression of cancer suppressor genes through targeting of epigenetic regulation. Takai *et al*(37) reported that HDACIs, such as vorinostat and valproic acid, were effective in six endometrial cancer cell lines. In this context, epigenetic regulation by microRNAs (miRNAs) is also important. In endometrial cancer, expression of a cancer suppressor-type miRNA, miR-152, has been shown to be inhibited by DNA hypermethylation. *DNMT1*, *MET*, *E2E3* and *Rictor*, all of which are associated with DNA methylation and cell proliferation, have been identified as target molecules in this process. These findings may potentiate the use of miR-152 in the treatment of endometrial cancer (38). Epigenetic mutations are clearly important in the initiation of carcinogenesis, particularly in type I endometrial cancer and further studies in this area are likely to be conducted.

The concept of molecular-targeted drugs is also likely to contribute to improvements in diagnosis. Over the last few years, numerous biomarkers of endometrial cancer have been identified. For example, Karaayvaz *et al*(39) demonstrated that the expression levels of miR-200c and miR-205 were significantly higher in endometrial cancer compared to normal tissues and that high levels of miR-205 were associated with poor prognosis. Identification and establishment of the properties of such biomarkers may permit objective and accurate preoperative diagnosis and prediction of prognosis, which currently depends mainly on histological techniques, allowing early the detection of endometrial cancer and selection of the appropriate treatment regimen.

## 6. Conclusion

Endometrial cancer is currently diagnosed based on tissue morphology and the treatment regimen is determined based on clinical findings and evidence from previous cases. However, it is becoming clear that mutations of genes associated with carcinogenesis underlie the various characteristics of cancer. Therefore, genetic and molecular information is increasingly being used for diagnosis, selection of treatment and prevention of cancer. The current response rates to molecular-targeted drugs as single-agent therapy are generally low and further trials are required, with a combination of currently available therapies and an investigation for factors determining sensitivity. Personalized treatment may be possible using an optimal molecular-targeted drug based on the overall gene expression profile of a patient, rather than the specific characteristics of the endometrial membrane. Thus, the concept and development of molecular-targeted drugs is likely to facilitate the next generation of personalized medical treatment.

## Figures and Tables

**Table I:** Response rates of molecular-targeted drugs.

Drug	Response rate (%)	Authors (Refs.)
Angiogenesis inhibitors		
Bevacizumab	13.5	Aghajanian *et al*(22)
Aflibercept	6.8	Coleman *et al*(24)
Thalidomide	12.5	McMeekin *et al*(25)
EGFR inhibitors		
Gefitinib	3.4	Leslie *et al*(26)
Erlotinib	4.3	Jasas *et al*(27)
HER2 inhibitors		
Trastuzumab	0.0	Fleming *et al*(29)
mTOR inhibitors		
Temsirolimus (first-line)	26.0	Oza *et al*(33)
Ridaforolimus	28.9 (CBR)	Colombo *et al*(34)

EGFR, epidermal growth factor receptor; HER2, human epidermal growth factor receptor type 2; mTOR, mammalian target of rapamycin; CBR, clinically beneficial response.
